# The Surname Space of the Czech Republic: Examining Population Structure by Network Analysis of Spatial Co-Occurrence of Surnames

**DOI:** 10.1371/journal.pone.0048568

**Published:** 2012-10-31

**Authors:** Josef Novotný, James A. Cheshire

**Affiliations:** 1 Department of Social Geography and Regional Development, Faculty of Science, Charles University in Prague, Prague, Czech Republic; 2 Centre for Advanced Spatial Analysis, University College London, London, United Kingdom; University of Utah, United States of America

## Abstract

In the majority of countries, surnames represent a ubiquitous cultural attribute inherited from an individual's ancestors and predominantly only altered through marriage. This paper utilises an innovative method, taken from economics, to offer unprecedented insights into the “surname space” of the Czech Republic. We construct this space as a network based on the pairwise probabilities of co-occurrence of surnames and find that the network representation has clear parallels with various ethno-cultural boundaries in the country. Our inductive approach therefore formalizes a simple assumption that the more frequently the bearers of two surnames concentrate in the same locations the higher the probability that these two surnames can be related (considering ethno-cultural relatedness, common co-ancestry or genetic relatedness, or some other type of relatedness). Using the Czech Republic as a case study this paper offers a fresh perspective on surnames as a quantitative data source and provides a methodology that can be easily incorporated within wider cultural, ethnic, geographic and population genetics studies already utilizing surnames.

## Introduction

The spatial distribution of surnames is far from random. Differences in early naming practices and unique regional, geographic, demographic, or migratory influences have led to considerable specificity with regard to mix of surnames that can be found in a particular place. Such specificity has been shown to capture a great deal of ethno-cultural variation that is often intertwined with the characteristics of an area [1]. In addition, surnames can often reveal aspects of large-scale population structure; for example, a good correspondence exists between changes in surname distribution and linguistic boundaries [2], [3], [4], [5], [6]. Given the paternal inheritance of surnames in many societies, surnames also have demonstrable utility as proxies for genetic information [7], [8], [9], [10], [11]. As has been demonstrated by [12], this offers enormous potential, especially in the context of developing more efficient sampling strategies in the context of population genetics.

Such applications of surname research are based on the key assumption that the spatial structure of surnames can, at least to some extent, mirror other aspects of population structure. To extract information from surnames, the challenge is to discern meaningful patterns from complex spatial distributions with little a priori information (generally related to ethnic categories). To our knowledge there has so far not been any attempt to capture the entire surname structure of a country through the pairwise comparison of geographic distributions of individual names. Previous research has ignored the spatial component altogether [13] or has been based on surname composition comparisons between administrative geographies [14].

This paper seeks to examine the surname structure of the Czech Republic (Czechia) by employing a suitable pairwise measure of relatedness between individual surnames based on their frequency of spatial co-occurrence in terms of their joint spatial concentration. This measure formalizes a simple assumption that the more frequently the bearers of two different surnames concentrate in the same locations the higher is the probability that these two surnames can be “related”. In this context, relatedness corresponds to surnames formed within the same community and those informed by similar cultural, ethno-linguistic or other factors. Using this measure, we depict the aggregate surname structure of Czechia as an undirected network of surnames linked by the degree of their relatedness. This representation can be conceptualised as “Czech Surname Space” and offers a template for similar research in other countries. Our inductive approach focuses on the revealed relatedness; only after the Czech surname space is determined do we map its structure and examine possible coincidences with other aspects of the Czech population differentiation.

## Materials and Methods

### Revealed relatedness between individual surnames

A focus on the spatial co-occurrence of surnames makes this paper distinct from previous studies. The bulk of the literature typically concerns pairwise comparisons between spatially defined populations based on the (di)similarity of their respective surname compositions [5], [6], [14], [15]. Here, we apply two modifications of the measure of pairwise relatedness used in very different context of the analysis of international trade [16]. These measures are novel in the context of surname analysis and we have found them to work better for our purposes than the traditional “genetic distance” measures such as Lasker or Neís indices (outlined in [17]).

The approach adopted here is a departure from previous research in the sense that the spatial distributions of individual surnames are the key input; regional patterns emerge as groupings in the surname space. Such approaches seek to establish the extent to which two or more geographic areas share the same pool of surnames and therefore offer comparisons between spatial units rather than the surnames themselves. With traditional methods, broad surname regions can be reliably produced but at the risk of subsuming some of the smaller groups of surnames with non-contiguous spatial patterning. Migrant surnames may, for example, be well-represented in these smaller groups and therefore more easily isolated than when using a traditional measure to produce more aggregate results. Improved granularity comes at the expense of increased computing overheads and a far more complex result (due to its larger number of comparisons), but we feel that capability to handle and interpret such outputs is increasing all the time and, as such, the methodology will become more widely applicable.

The first step in defining a surname spatial similarity measure is the selection of an appropriate form of input data for describing the occurrence of individual surnames in particular regions. A simple consideration of the absolute numbers of bearers would be inappropriate in the present context because the size of subpopulations of individual surnames varies immensely. A better metric that accounts for both the spatial concentration and the ubiquity of individual surnames is the location quotient (*LQ*). For individual surnames (*i*) and regions (*r*), respectively, it can be expressed as:
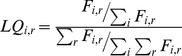
(1)where *F_i,r_* stands for the absolute number of bearers of the surname *i* in the region *r*. The *LQ_i,r_* compares the relative share of people with the surname *i* in the population of the region *r* relative to the share of this surname in the whole population at a more aggregate level. An *LQ_i,r_* >1 indicates that the surname in question is more prevalent in the region *r* than in the whole population (below we simply say that the surname concentrates in the region *r*).

In the second step, the *LQ* is used for the expression of the pairwise measures of revealed relatedness between surnames. For this paper the Jaccard and Dice similarity measures were examined. Here the Jaccard establishes the number of regions where both of the two analyzed surnames are concentrated relative to the number of regions where at least one of them concentrates. The Jaccard measure of the revealed relatedness between the two surnames *i* and *j* when focusing on their co-occurrence over *r* regions is defined as:
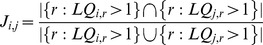
(2)where the nominator accounts for the number of regions that satisfy both *LQ_i,r_* >1 and *LQ_j,r_* >1, while the denominator refers to the number of regions satisfying at least one of these inequalities. The measure falls between 0 and 1 with the upper bound signifying that the two surnames in question are concentrated solely in identical regions.

In this context, the first asymmetric Dice measure captures the probability that surname *i* concentrates in the region *r* conditional to the concentration of surname *j* in the same region:
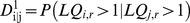
(3)

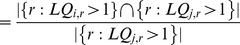
(4)


Similarly, the second Dice measure calculates the probability that surname *j* concentrates in the region *r* conditional to the concentration of surname *i* in the same region:
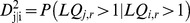
(5)

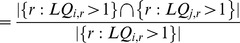
(6)


For the present purpose we need a symmetric measure of relatedness and thus consider the smaller from the two asymmetric Dice measures presented above. As such, we define the symmetric Dice measure of revealed relatedness between the surnames *i* and *j* as:
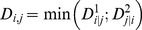
(7)


The appropriateness of the above defined Jaccard and Dice measures has not been tested with surname data. We therefore sought to establish the possible impacts of differing population sizes of individual surnames. We undertook a number of Monte Carlo simulation tests to establish the properties of the indices in this respect (Text S1). We found that the Dice coefficient is slightly less sensitive to the size differences and more stable in terms of smaller fluctuations in results obtained from repeatedly generated pseudorandom data. In general we have noted that both of the measures can serve well for our purposes and we undertook all of our calculations for both the indices. However, because of space limitations, the graphical results presented and their associated analysis use the Dice coefficient only. Given the specification of our analysis described below, the sets of surnames linked by the 50,000 highest pair-wise observations of Jaccard and Dice measures, respectively, calculated at the more detailed level of municipalities (that is where a higher discrepancy may be expected) are 80% identical.

### Constructing the surname network

Given the large sample of surnames analyzed it was necessary to run a series of computationally intensive calculations to obtain an extensive matrix of surname-surname proximity observations (nearly 200 million in the first stage of our analysis as described below). Such a matrix tends to be very sparse with a large number of zero or negligible observations and very few more significant observations. It is therefore conducive to data mining through network analysis (the matrix can also be referred to as the weighted adjacency matrix as in [18]). We thus consider the network of surnames in terms of an undirected graph where nodes (or vertices) correspond to individual surnames and links (or edges) between them refer to the most significant measures of revealed relatedness (*D_i,j_* has been applied for the results presented below). As stated above, we consider this network as an appealing representation of the Czech surname space. It can be examined both globally in terms of its aggregate patterns, its shape, or the number of communities, and locally through extracting the positions of individual surnames or their groups. Both of these aspects are important with respect to our inductive analysis that is driven by an expectation of detectable clusters or communities and surnames with strong internal and relatively weak external relatedness.

For the network visualization we used Cytoscape, open source software suitable for handling large complex networks [19]. A force-directed algorithm with consideration of weights linearly proportional to our measure of revealed relatedness appeared to produce the most effective network layout (for description of the force-directed layout used in Cytoscape software see http://cytoscapeweb.cytoscape.org/documentation/layout). With this the network can be conceptualised as a physical system where nodes (surnames) influence each other via attracting forces with strengths proportional to their revealed relatedness. The algorithm minimizes the energy of the physical system and assigns the nodes with positions in two-dimensional space accordingly.

For the network visualisation to be interpretable, the majority of negligable links should be removed. A threshold of *D_i,j_* (denoted as *d*) determined by, for example, inspecting the frequency distribution of the proximity observations provides a logical criterion. Considering a certain *d*, a surname space visualisation consists of *n* surnames and *m* surname-surname relatedness links, when:
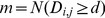
(8)

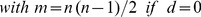
(9)


This provides the basis to defining some simple local and global characteristics of the surname network, similarly to basic measures used in the network analysis [18]. An important local parameter pertaining to each node is the node degree. It is the number of links that connect the node in question to other nodes in the network. Here the degree of a surname *i* is denoted as *k_i_* and it corresponds to the number of its revealed relatedness links to other surnames equal or above chosen *d*:
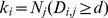
(10)


This measure is particularly interesting in the present context because it can be considered as a simple measure of the node centrality. A high *k_i_* implies that surname in question co-occurs (concentrates in similar regions) with many other surnames within a given surname space or its sub-space. In other words, a high *k_i_* indicates that a surname *i* is highly embedded in the surname space or its sub-space (which is understood here as any contiguous part of the surname network, defined for example by a selection of adjacent nodes or links) and that it can be considered an examplar of a local population.

In addition, two basic global parameters of a surname space can be introduced in terms of the mean surname degree (*c*) expressed as:
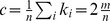
(11)and the surname space density (*ρ*): that is the proportion of actual number of links in the surname space relative to the maximum possible number of links:



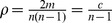
(12)Both *c* and *ρ* are valuable metrics measuring the extent of aggregate relatedness among surnames within a given surname space (or its sub-space). As such, they provide interesting information about the extent of internal population homogeneity.

### Data and analysis design

This paper draws on a unique dataset containing the occurrence of individual surnames in each of the 6,253 Czech municipalities derived from the 2009 Central Population Register (produced by the Czech Ministry of the Interior). The data cover all those with permanent residence; that is Czech nationals and foreigners staying on a long-term basis. The 10,705,763 individuals listed share 362,125 unique surnames.

It is conventional in Czechia to have male and female variants of the same surname. Both exhibit almost identical spatial distributions negating the need to include both forms and so the female variants were omitted. This dramatically reduced the volume of data. Fortunately, nearly all Czech feminine derivatives are easily distinguishable by the suffix “–á”. The exceptions are comparatively more frequent for certain surnames typical of eastern Moravia and Silesia [20] and among rare surnames (see Table 1). Although a few of these exceptional cases have been included into the analyzed sample, it does not have any significant effect on results because the location quotient (as described above) compares relative population shares.

**Table 1:** Frequency distribution of all surnames and feminine derivatives with suffix “–á”.

Size category:	>9,999	>999	>99	>49	>9	>2	All
Nm. of surnames	33	1,379	17,210	30,307	88,376	185,121	362,125
Nm. of bearers	535,693	3,748,590	7,832,685	8,750,015	10,003,339	10,482,187	10,705,763
Share of bearers in total population	0.050	0.350	0.732	0.817	0.934	0.979	1.000
Nm. of feminine derivatives with suffix “á”	17	704	8,531	14,820	40,841	78,596	140,732
Share in number of all surnames	0.515	0.511	0.496	0.489	0.462	0.425	0.389

For the analysis of co-occurrence we decided to work with male surnames with a frequency exceeding 49 bearers in the whole country. With this filter applied the data comprised 15,487 most frequent male surnames and 4,347,283 individuals corresponding to 83% of total male population. The cut-off was chosen in the light of the following: firstly, the size distribution of surnames is heavily right skewed and the inclusion of less frequent names would make our analysis excessively computationally intensive (as described below); secondly, and more importantly, the consideration of less frequent surnames would considerably increase a risk of contamination of results by random co-occurrences of rare surnames; thirdly, we also noted that the spatial distribution of rare surnames in Czechia is quite uneven with significantly higher shares of such surnames in peripheral areas and especially in the region of Silesia (basic information about regional division of the country and main migratory processes that have shaped its current ethnic structure is provided in Text S2 and Figure S1). Therefore, the inclusion of rare surnames would disproportionately enlarge the parts of surname space that depict surnames concentrated in these regions.

As previously noted, the scope of the proposed study has been constrained by the computational intensity of the analysis and the nature of Czech administrative geography in this context. Initially, we attempted the analysis directly at the finest spatial level of municipalities. However, these spatial units were too fragmented and differing in population sizes to the extent that small numbers became an issue. Instead, we opted for a two-stage procedure (Table 2). In the first stage we undertook the analysis using a set of larger spatial units corresponding to 206 micro-regions (so called municipalities with extended competence). Importantly, the delineation of these units coincides relatively well with historical and socio-economic processes and they can be considered as functional socio-geographical micro-regions. The first stage of our analysis highlighted a smaller sub-sample of “important” surnames in terms of those most frequently co-occurring over these regions. As described in Table 2 and discussed in more detail below, in this way 5,660 of the potentially most interesting surnames (that is 36% of the original sample equivalent to nearly half of the male population) linked by the most significant pairwise measures of relatedness were identified. This set of surnames was then analyzed in the second stage of our analysis focusing on co-occurrence in 6,244 municipalities (the original set of municipalities contained 6,253 units but in nine of them none of 5,660 surnames indentified in the first stage of our analysis is concentrated). This approach is based on the assumption that the pairs of surnames with high co-occurrence in larger regions will also have a higher probability of being found together in smaller regions. For the second stage, the three largest municipalities (in terms of population size) including Praha, Brno, and Ostrava were excluded from the analysis as we expect many “random” co-occurrences to be found, thus adding noise to the results.

**Table 2:** Description of samples of surnames and spatial units in the first and second stage of analysis.

	Surnames			Spatial units		
	Nm. in sample	Of all male surnames	Of total male population	Nm.	Average pop. size*	Median pop. size*
1^st^ stage	15,487	7%	83%	206	21,103	12,504
2^nd^ stage	5,660	2.5%	48%	6,244	347	109

* Refer to individuals bearing surnames included in the analysed samples of surnames.

We expect that the consideration of co-occurrence indices in more aggregate spatial units can provide us with a “global” picture, whilst analysis at the level of municipalities will lead to more fragmented network identifying more accurately the pairs and communities of individual surnames with the highest probability of being factually related.

## Results

### Analysis of co-occurrence in 206 micro-regions

**Figure 1:**
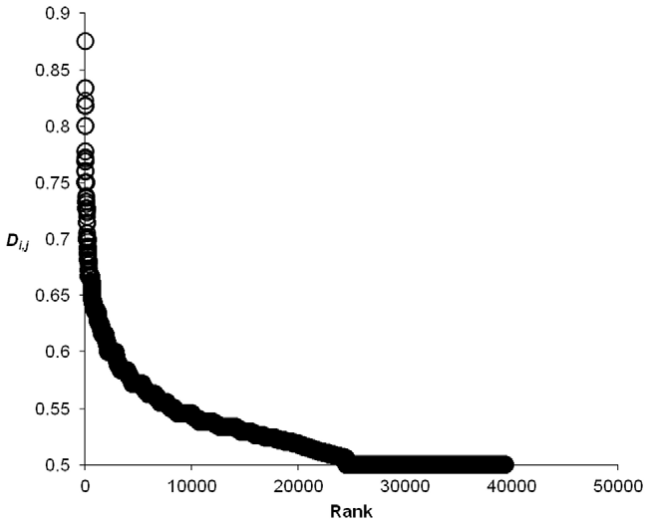
Rank-size distribution for the set of observations with *D_i,j,reg_* ≥0.500.

We first examined the co-occurrence of 15,487 unique male surnames over 206 Czech micro-regions. The calculations for all possible pairs of these surnames produced a matrix of 119,915,841 proximity observations (*D_i,j,reg_*). Table 3 shows the upper part of cumulative frequency distribution for these results and Figure 1 depicts its rank-size distribution. As expected, the frequency distribution is heavily skewed to the right with only 0.13% of all *D_i,j,reg_* observations attaining a value exceeding 50% of the maximum observation. In other words, while an overwhelming majority from all of analyzed pairs of surnames reveal a negligible relatedness, there is also a tiny proportion of those pairs that are interesting in the present context because of their high mutual proximity.

**Table 3:** Upper parts of the cumulative frequency distributions of *D_i,j,reg_.*

	Bounds in % of maximum observation					
	= 100%	>90%	>80%	>70%	>60%	>50%
Number of proximity links	2	28	233	1931	16759	159137
Number of surnames	3	30	116	512	4388	12828
Male population covered	0%	1%	2%	7%	41%	77%

The maximum observation corresponds to *D_i,j,reg_*  = 0.875. Based on 119,915,841 observations of *D_i,j,reg_* between 15,487 surnames.

Before examining the most significant links it is worth discussing some of the highly ubiquitous surnames in terms of greater prevalence but low spatial concentration. The following six surnames have more than 4,000 bearers but have no observation exceeding *D_i,j,reg_* of 0.500: Hruška (means a pear in English), Hrubý (originated from older term for tall), Liška (a fox), Toman (after Thomas the Apostle), Kočí (a coachman), Prokop (probably from Greek prokoptó or prokópos meaning pioneer and ready, respectively). The ubiquity of these and similar surnames is determined by a meaning independent of regionally specific naming practices (a common naming practice in many countries). Of the 675 male surnames with the frequency above 1,000 bearers only 17% of them fall into this group of spatially ubiquitous surnames. Importantly, it implies that the majority of the most frequent surnames exhibit some kind of spatial concentration. Arguably, the tendency towards spatial concentration is expected to be even higher for the less popular names.

For this paper, the most interesting information is contained in observations pertaining to the steep left part of the rank-size plot in Figure 1. On inspection, we found that the value of *D_i,j,reg_*  = 0.525 (or 60% of the maximum) offers a good threshold for distinguishing these important observations as it lies in the area beyond which the rank-size curve rapidly flattens. Using the conditional probability interpretation of the Dice coefficient, we can say that two surnames connected by a link satisfying *D_i,j,reg_* ≥0.525 have at least 52.5% probability that one of these surnames concentrates in a region where another is concentrated.

**Figure 2:**
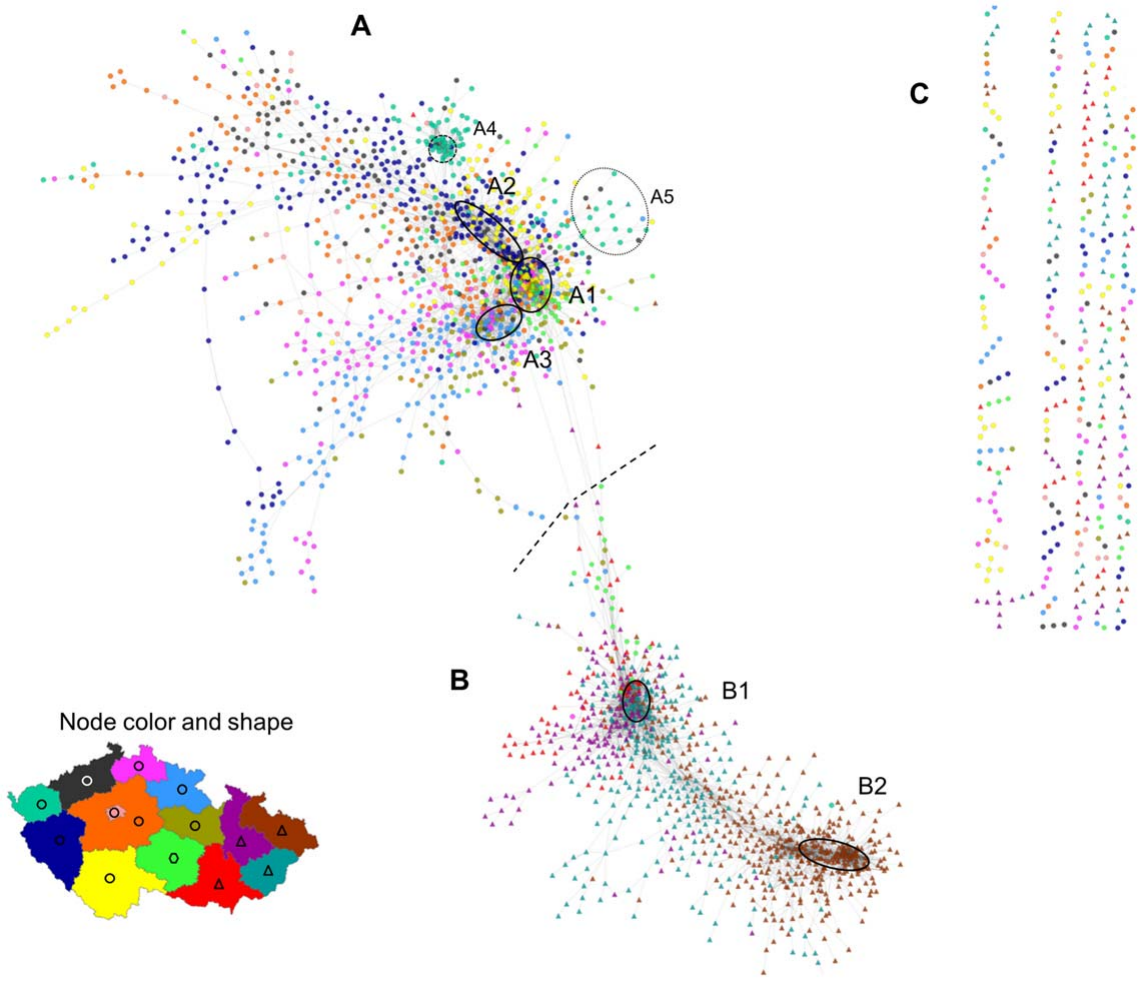
Czech surname space based on the analysis of co-occurrence in 206 micro-regions. A – Bohemian part of the surname space; B – Moravia-Silesia part; C – Smaller communities and pairs of surnames disconected from the main network (surnames with links *D_i,j,reg_* <0.500 to all of the surnames in the parts A and B but with Di,j,reg ≥0.500 to one or more surnames in part C). Dashed line indicates approximate separation between parts of the surname space pertaining to Bohemia and Moravia-Silesia. A1-5 and B1-2 indicate main core communities of surnames (as described below in the text). The color and shape of a node is determined on the basis of the region (14 administrative regions known as “kraje” or NUTS3 regions were used) where the surname has the maximum concentration (max *LQ_i,r_*). Circular nodes show surnames with maximum *LQ_i,r_* in a Bohemian region, triangles mark surnames with the maximum *LQ_i,r_* in a Moravian or a Silesian region, and hexagons are used for surnames with the maximum *LQ_i,r_* in Vysočina region which is partly in Bohemia and partly in Moravia. See Figure S2 for a high resolution version with labels of individual surnames and the size of nodes scaled by their population size and Figure S3 for a high resolution version where the size of nodes refers to their degree.

Unfortunately, despite this cut-off, the surname space still contained too many nodes to be reasonably visualised. We thus further limited the displayed results to surnames with at least 100 bearers. This value is based on insights from a number of preliminary experiments examining the trade-off between the number of surnames displayed (complexity of displayed surname network) and graphical limitations of our network visualisations (readability of the network). As a result, we obtained a set of 8,405 proximity links connecting 2,429 unique male surnames. After applying the weighted force-directed layout algorithm, the aggregate version of the Czech surname space was generated and visualized in Figure 2 (see Figure S2 for a high resolution figure where the nodes are labelled and their size is scaled by their population size and Figure S3 for a high resolution version where the size of nodes is scaled by their degree).

The Czech surname space illustrated in Figure 2 consists of the bulk of nodes comprising two clearly distinguishable parts (A and B) and a number of smaller communities and pairs of surnames disconnected from this main network (marked as C in Figure 2). The majority of the network aligns surprisingly well with the division of the country into three historical lands (Bohemia, Moravia, Silesia – see Text S2 and Figure S1) that can be considered as the main historical population regions of Czechia. The larger upper part of the surname space (A) contains surnames concentrating and co-occurring predominantly in Bohemian regions, while the smaller lower part (B) consists mainly of Moravian and Silesian surnames. Comparing the mean node degree and network density between these two components of the Czech surname space (Table 4) suggests a greater aggregate relatedness within the Moravian-Silesian part. This indicates more stability of Moravian and Silesial population relative to its Bohemian counterpart. Again, this aligns well with what can be expected when taking the cultural and historical specifics of Czechia into account.

**Table 4:** Basic characteristics of Czech surname space in Figure 2 and its main parts.

Part of the surname space	Number of surnames (*n*)	Number of links (*m*)	Mean surname degree (*c*)	Density (*ρ)*
A – Bohemian	1200	4315	7.2	0.006
B – Moravian-Silesian	877	3885	8.9	0.010
C – disconnected communities	352	205	0.7	0.001
Czech surname space total	2429	8405	6.9	0.003

**Figure 3:**
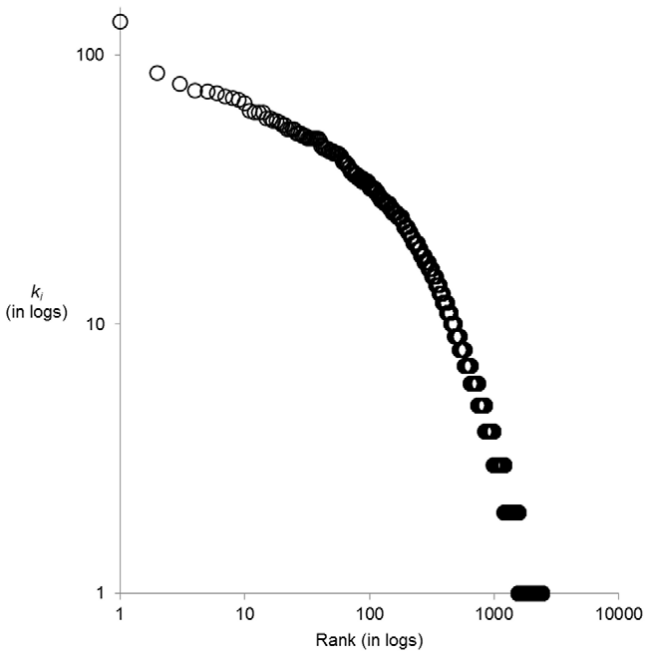
Degree distribution of surnames in the Czech surname space (as displayed in Figure 2).

**Figure 4:**
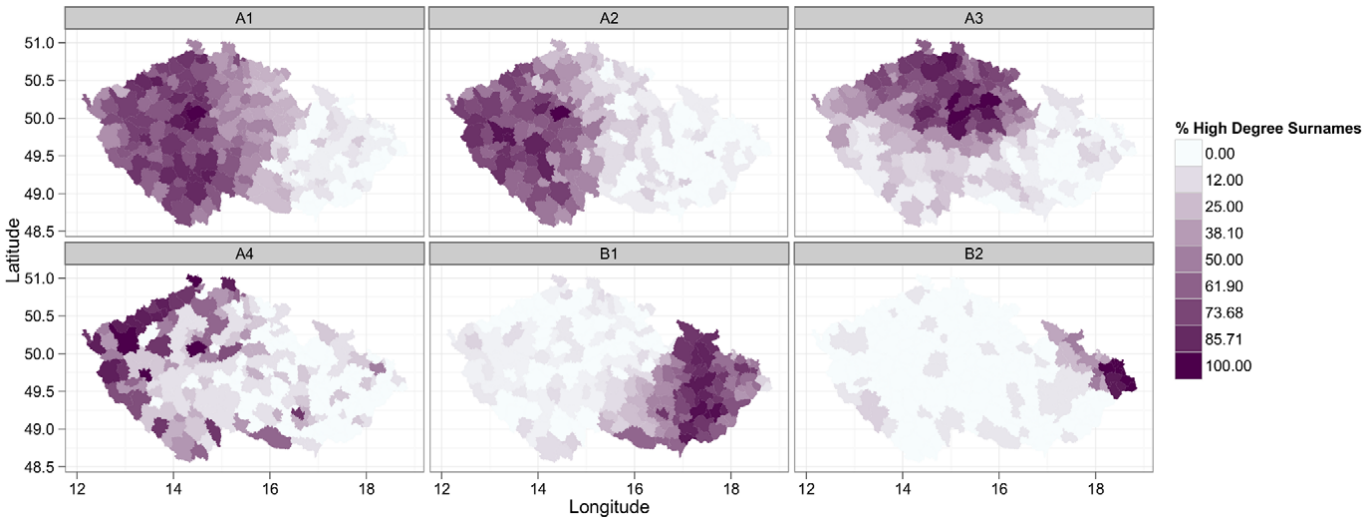
Spatial concentration of individual communities of high degree surnames. Individual maps show regional variation in the percentage of high degree surnames from particular core communities (A1, A2, A3, A4, B1, B2) as listed in Table 5 concentrated in a given region. For example, if the percentage of high degree surnames for A1 (the upper left map) corresponds to 100, then all the surnames listed in the A1 group in Table 5 are concentrated in a given region (that is, all of them satisfy *LQ_i,r_* >1 for the region in question).

The key feature of each network graph is its degree distribution. In a random graph, nodes have a similar probability of being connected and therefore the degree distribution tends to be homogenous as signified by a binomial shape. By contrast, real world networks of various complex phenomena are typically hierarchically organized, with an inhomogeneous, considerably right skewed degree distribution. Here, a highly inhomogeneous degree distribution has been found (Figure 3) suggesting that the Czech surname space depicted above may share some general properties of complex networks. While a few surnames reveal many significant links to other surnames, a majority of them have a negligible number of these significant links. In addition, as is clearly visible in Figure 2, our network is also globally inhomogeneous in the sense that high degree nodes are not distributed evenly but clustered into a few dense communities. We are particularly interested in the highest degree hub surnames within the core clusters as they are the most embedded within the Czech surname space, and they can be regarded as the most typical exemplars. In addition, we are similarly interested in the identification of surnames outside the main cores that still have a high degree relative to other peripheral surnames and that serve as secondary hubs. These are regionally important exemplars, which, together with the highest degree surnames, form a “back-bone” of the Czech surname space. Both types of these hub surnames are listed in Table 5 when classified into several regionally specific groups (as described below). High resolution Figure S3 then maps the exact position of high degree surnames within the surname network, while showing variation in the degree of particular surnames by different node sizes. In addition, Figure 4 shows regional concentration of these high degree groups of surnames from particular core communities as listed in Table 5. Interestingly and importantly, we found that there is a lack of relationship between the surname degree and its frequency of occurrence (Figure S4). It contrasts with a naive expectation that the highest degree surnames will predominantly be the most frequent ones, while less frequent surnames will automatically reveal a low node degree.

**Table 5:** High-degree nodes in particular parts of Czech surname space as indicated in Figure 2 (*k_i_* in parentheses).

Community (as indicated in Figure 2)	Highest degree surnames in particular groups
A1	Mašek (78); Kohout (74); Bláha (72); Soukup (70); Tůma (62); Šindelář (57); Zelenka (56); Mareš (55); Novák (51); Vacek (50); Hora (49); Nedvěd (49); Průcha (49); Šíma (46); Mařík (46); Černý (45); Šašek (44); Brož (43); Čapek (43); Pech (43); Kouba (42); Jindra (41)
A2	Levý (53); Fencl (49); Nový (44); Čadek (40); Fořt (37); Sloup (37); Zíka (35); Hořejší (28); Větrovec (28); Houška (22); Hůrka (22); Krákora (22); Voráček (21); Bečvář (21); Jindřich (20); Kuneš (20); Vácha (20)
A3	Klouček (61); Jirásek (53); Šulc (50); Vondráček (45); Janata (44); Krejčík (43); Šimůnek (40); Hanuš (30); Matouš (29); Stránský (28); Krupička (28); Bartoníček (28); Kout (25); Kopecký (25); Chvojka (24); Horyna (23); Bareš (22); Pilař (20); Zima (20)
A4	Nguyen (44); Nguyen Thi (39); Pham (36); Vu (30); Tran (29); Nguyen Van (26); Dinh (25); Dang (25); Le (23); Bui (20)
Other regional hubs in Bohemia	Šmejkal (31); Hříbal (26); Douša (26); Kasl (24); Duchek (18); Drbohlav (17); Třešňák (17); Vinš (16); Salač (16); Trejbal (16); Švandrlík (15); Sucharda (14); Štýbr (12); Mádle (12)
B1	Polášek (133); Zapletal (86); Přikryl (73); Konečný (69); Hanák (66); Večeřa (58); Janík (53); Klimek (53); Hradil (51); Machala (51); Chovanec (49); Sedlář (49); Vaculík (48); Zbořil (44); Polách (43); Vala (40); Buček (37); Doležel (37); Chytil (36); Jurečka (34); Zlámal (34); Zaoral (33); Blaha (32); Navrátil (32); Tomeček (32)
B2	Sikora (68); Kawulok (61); Kubiena (61); Lysek (58); Kajzar (57); Valošek (55); Ligocki (50); Spratek (49); Pawlas (45); Liberda (45); Byrtus (43); Walach (42)
Other regional hubs in Moravia-Silesia	Strnadel (49); Zátopek (40); Ondruch (29); Kresta (25); Šrubař (23); Petroš (23); Juchelka (22); Kocurek (21); Maléř (18)

The most extensive cluster of surnames in the Bohemian part of the Czech surname network in Figure 2 forms its primary core. Whilst the core is clearly recognizable upon the visual inspection of the graph based on a force directed layout, our effort to define it more precisely through the application of community detection algorthms failed to offer a better solution. Reassured by the way the core clearly delineates a known population boundary when its surnames are mapped and with the help of the prevailing regional concentration of individual surnames (visualized by different node colours in Figure 2), we distinguished three different groups of surnames within this main Bohemian cluster. For each surname *i*, the region of its prevailing concentration refers to a region with the maximum *LQ_i,r_* (here we considered 14 administrative regions known as kraje or NUTS 3 regions using the terminology of the EU Nomenclature of Territorial Units for Statistics). The three distinguished groups within the main Bohemian core were indicatively marked as A1, A2, and A3 in Figure 2.

The group A1 includes typical Bohemian surnames in terms of the most frequent (the three most common are Novák, Svoboda, and Novotný) in addition to other lower frequency, and traditional, Bohemian surnames. Although the more populous of these surnames are widely found across the country, all of the surnames from this group tend to be concentrated in the south and west regions of Bohemia (see also Figure 4). The second group within the core cluster of Bohemian surnames (A2) is partially overlapping with the first one, while containing typical south-west Bohemian names. By contrast, the third group (A3) consists of surnames typically found in the north and north east of the Bohemia region. The separation of this community from the two previously mentioned is recognizable and it also holds for their respective peripheries.

In addition to the main core, there is another dense cluster in the Bohemian part of the Czech surname network. Labelled as A4 in Figure 2, it contains a community of Vietnamese surnames. It results from a significant spatial concentration of Vietnamese immigrants and their descendants in the western and particularly north-western regions at the border with Germany and also big cities [21], [22].

**Figure 5:**
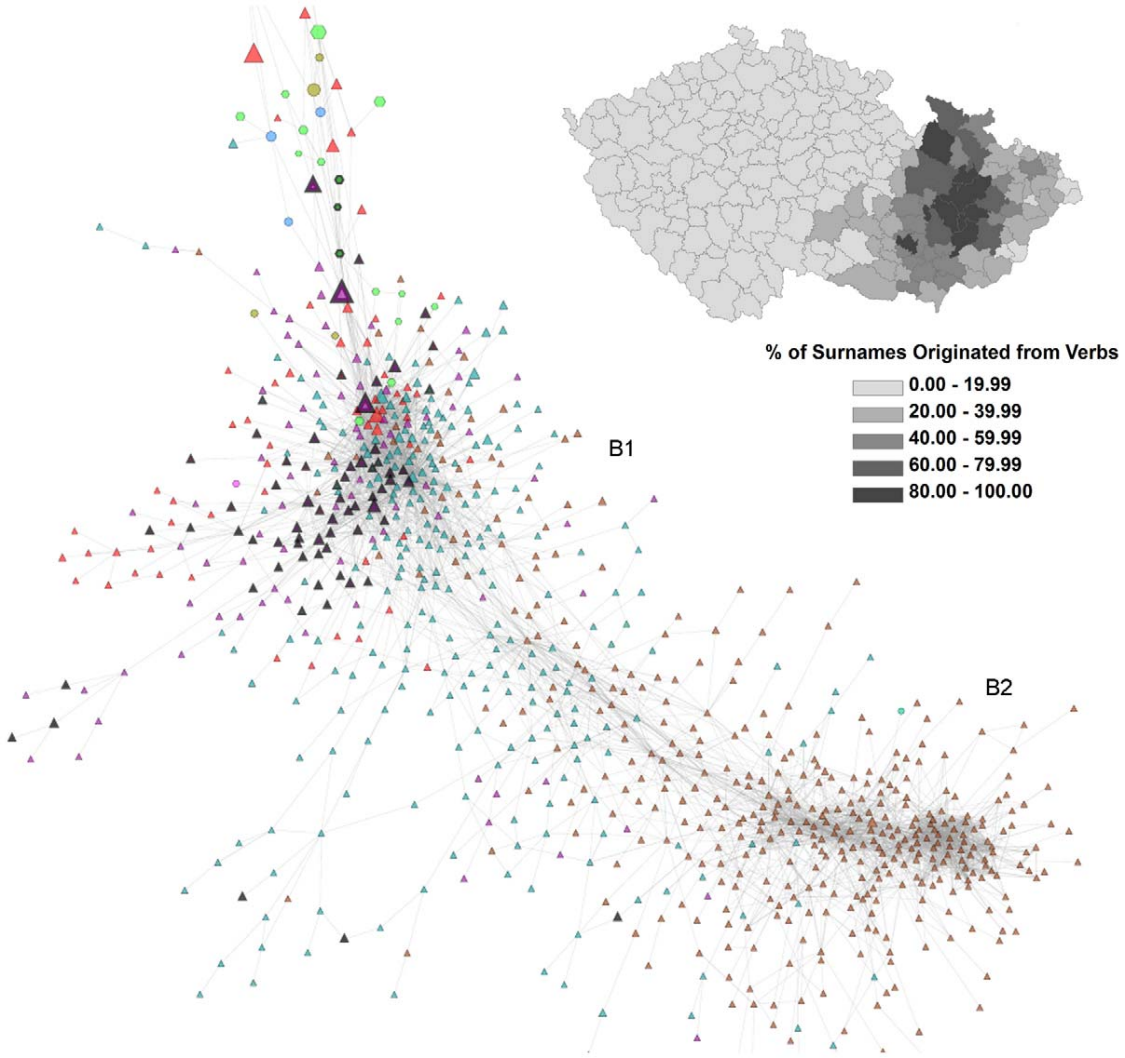
Surnames originating from verbs. The nodes pertaining to surnames that have originated from verbs are marked by the black bold node borders. The surname network corresponds to the B part of the Czech surname space as displayed in Figure 2. The map shows regional variation in the percentage of the surnames originated from verbs concentrated in a given region (70 “verbal surnames” indicated in the network were considered).

The second Moravian-Silesian part of the Czech surname space has two dense cores in terms of the main Moravian cluster (B1) and Silesian cluster (B2). In addition, there is also a relatively dense area between these main cores consisting of names typical for various more specific regions in the north, central, eastern Moravia. In the case of Moravian surnames, linguistic differentiation of surnames and spatially specific naming practices are clearly recognizable. For example, a majority of names that apparently originated from verbs (most often these surnames are in a past conditional form of a verb) are located in the lower left and upper parts of the main Moravian cluster (B1). Some notable examples of these names, with a quite central position in our surname network (see below), are Zapletal (past conditional from “to weave”), Přikryl (from “to cover”), or Hradil (from “to block”). In Figure 5 the position of nearly 70 of such surnames identified within this part of the Czech surname space is indicated by the black bold borders of their respective nodes. The figure also contains the map showing the spatial concentration of these naming practices to certain specific regions of Moravia.

**Figure 6:**
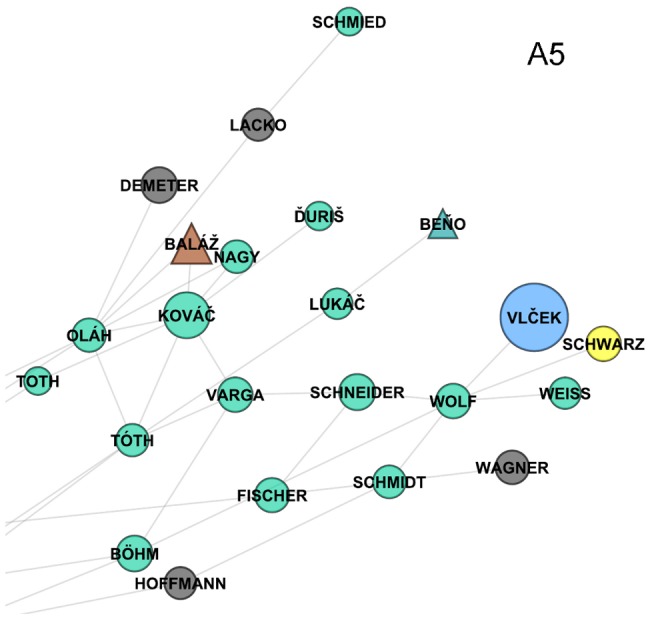
Peripheral communities of German and Roma surnames (area in Figure 2 labelled as A5).

In addition, Figure 6 shows another smaller but quite interesting group of surnames located next to each other at the very right edge of the Bohemian part of the Czech surname network (the area indicated as A5 in Figure 2). These are typical Roma surnames (the upper left part of Figure 6) and some German origin surnames (the lower right side). These surnames are concentrated in the same regions along the western and northern border of the country, which is a part of so called Sudetenland, and the similarity in their spatial behaviour can be linked to some disruptive population changes that affected these areas after the Second World War. The identification of German surnames can be seen as relicts of significant share of German population that had been living in these regions for centuries until their post-war expulsion (Text S2 and Figure S1). The Roma surnames can be then interpreted as a result of the subsequent resettlement and industrialization led immigration into these areas, but also of some state policies that have contributed to the spatial concentrations (and often also segregations) of Roma minority groups [23].

An intriguing exception to these explanations is a typical Czech surname Vlček that can also be found in Figure 6 because of its significant revealed relatedness with Wolf. From all of the surnames considered, the name Wolf has been found as the nearest neighbour of Vlček, with the 56.7% probability that one of these surnames concentrates in the region where another one is concentrated. The high co-occurrence of these two surnames in the identical regions seems to be attributable to their common meaning – Vlček literally means “small Wolf” in the Czech language. The bi-lingual naming practices or secular name transformations taking place in these historically multi-ethnic regions (German and Czech) offers the most likely explanation for such commonalities.

### Analysis of co-occurrence in municipalities

In the second stage of our analysis we examined the co-occurrence of Czech surnames at the finest spatial level of 6,244 municipalities. We began with the calculation of the pairwise indices of revealed relatedness (*D_i,j,mun_*) among 5,660 surnames selected on the basis of the highest revealed relatedness at more aggregate spatial level. This sample of surnames covers almost a half of the Czech male population. Given the significantly higher number of spatial units considered for this second stage of our analysis, the values of *D_i,j,mun_* are generally lower than *D_i,j,reg_* in the first stage which focused on co-occurrence in 206 micro-regions only. At the same time, the size distribution of these second stage results is even more skewed to the right; the maximum *D_i,j,mun_* (from the total of more than 32 million of observations) corresponds to 0.687, while only 0.011% of all observations exceed 50% of the maximum value. These differences between the first and second stage results are understandable and go hand in hand with the expectation that the surname network based on the municipality level calculations will be more fragmented.

**Figure 7:**
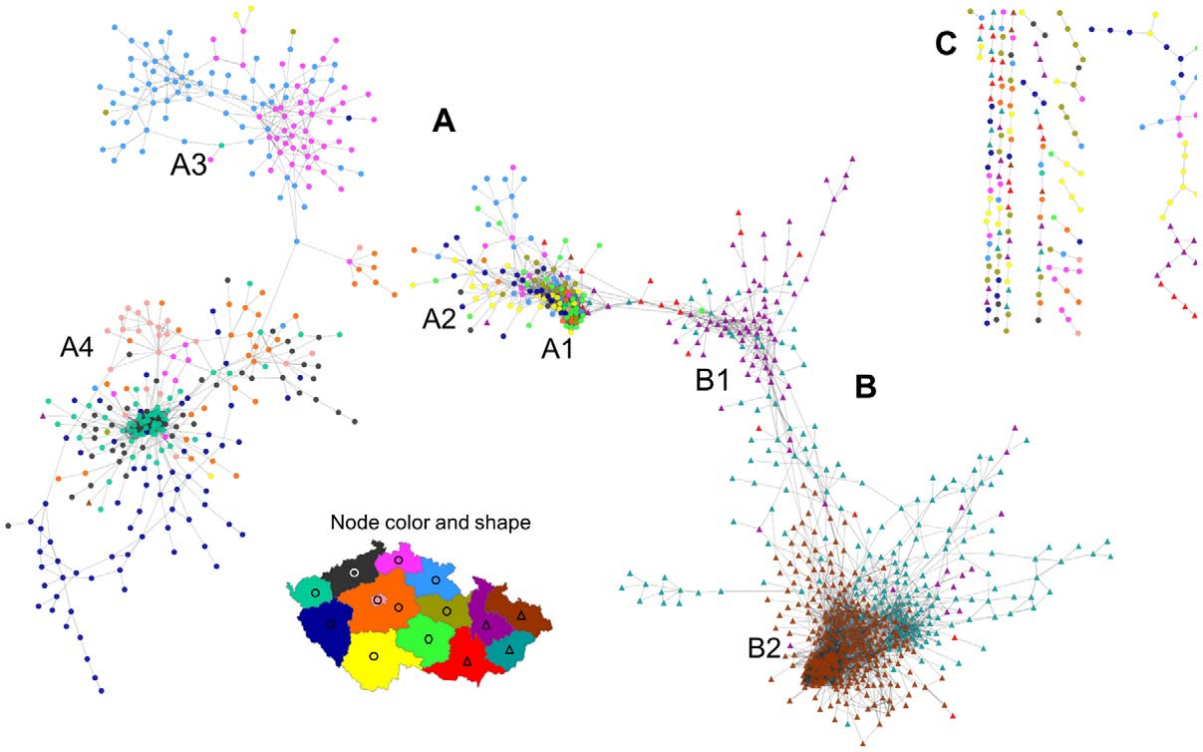
Czech surname space based on the analysis of co-occurrence in 6,244 municipalities. A – Bohemian part; B – Moravian-Silesian part; C – Smaller comunities and pairs of surnames disconected from the main network. A1-5 and B1-2 indicate core communities of surnames. The color and shape of a node is determined on the basis of the region (14 administrative NUTS 3 level regions were used) where the surname has the maximum concentration (max *LQ_i,r_*). See Figure S5 for a high resolution version with labels of individual surnames and the size of nodes scaled by their population size.

This has been confirmed by the fact that a majority of the most significant *D_i,j,mun_* proximity observations occur among relatively rare surnames that are typically concentrated in a few nearby municipalities. This is especially the case of Silesian surnames that account for almost all *D_i,j,mun_* observations at the very top of the distribution of results. As such, in order to get a reasonable network representation, we again had to impose some restrictions in relation to the minimal size of surnames shown as nodes and the strength of links between them. After applying the criteria from the previous section, we found the frequency of at least 150 bearers and the links determined by *D_i,j,mun_* >0.23 to be optimal. The surname network based on these parameters and generated by a weighted force-directed algorithm is depicted in Figure 7 (Figure S5 depicts a high resolution version with labels of individual surnames and the size of nodes scaled by their population size).

In general, the second stage or municipality level surname network has reproduced the macro-division of the Czech surname space identified in the first stage and described above. The proportions between the sizes of the main clusters are however different with the previously mentioned dominance of the dense group of Silesian surnames (B2). Regarding Moravian surnames, again the commonality of verb-derived surnames emerges, as they form the majority of names in the B1 area of the network. The Bohemian part of the surname space (A) is structured into three main groups of surnames. The A1 cluster comprises some of the most frequent surnames and those prevalent across most of Bohemian regions, whilst the separation from the secondary cluster (A2) is hardly discernible. By contrast, two other core areas are well recognizable and represent northern and eastern Bohemian names (A3) more specifically and surnames concentrated mainly in municipalities in the north-west and west of Bohemia (A4).

The general congruence in macro-structure of the surname networks constructed here and in the first stage of our analysis is an important finding (generally similar macro-structure was also found when the *J_i,j,reg_* and *J_i,j,mun_* were considered instead of the *D_i,j,reg_* and *D_i,j,mun_*, respectively). However, the main value of this second stage municipality level exercise should be seen in individual details uncovered with respect to local parts of the surname network. A number of interesting examples of pairs of surnames that have been found as potentially closely related, regionally specific offshoots of the surname network, or specific groups of surnames determined in various ways, could be identified, mapped, and examined in greater depth.

**Figure 8:**
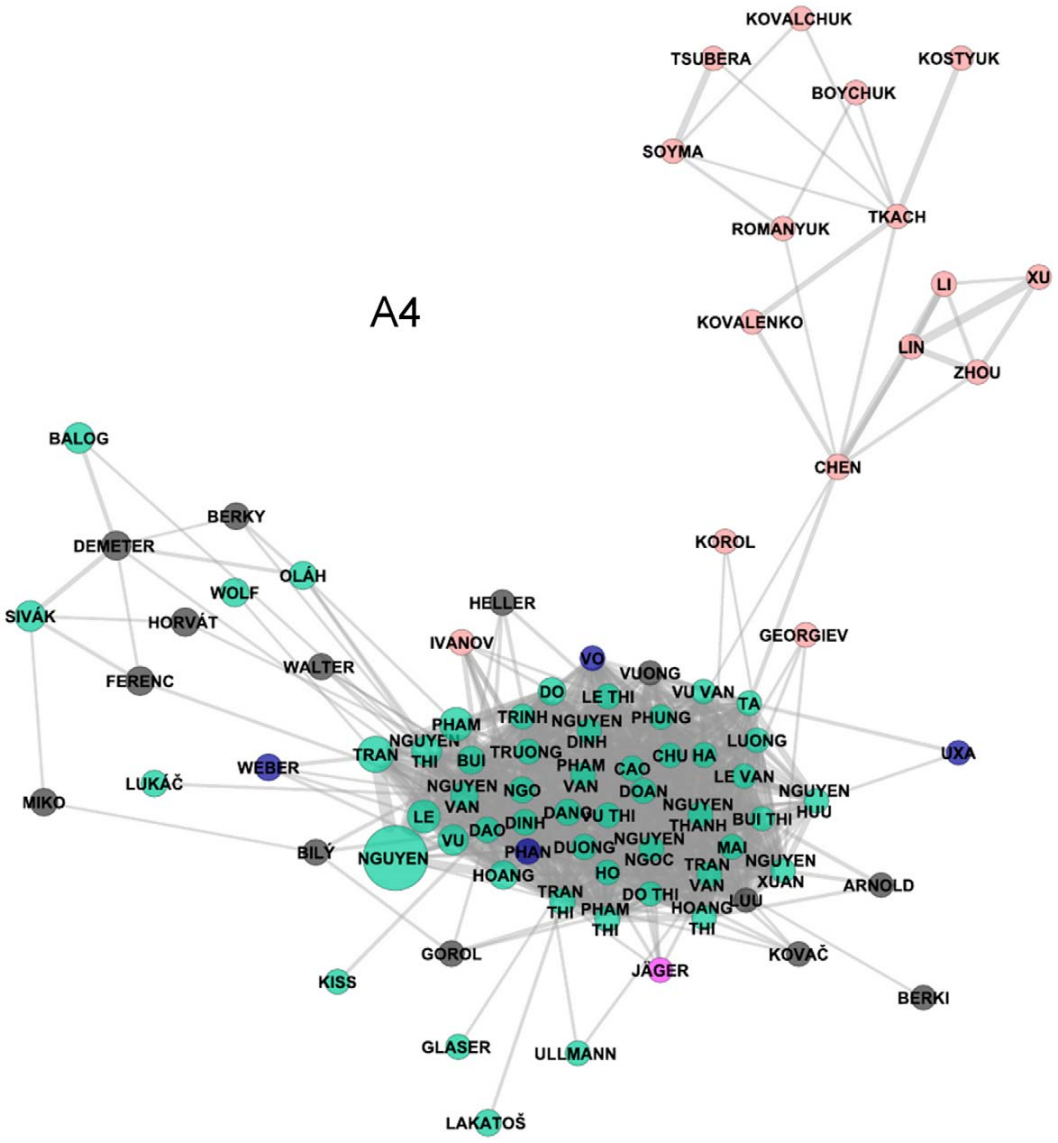
Cluster of Vietnamese surnames and their “surroundings”.

For example, Figure 8 illustrates the applicability of the approach for the classification of population into ethnic groups and the subsequent indication of the degree of relatedness both within identified groups and outside them. It offers a closer look at the surroundings of the dense cluster of Vietnamese surnames (indicated as A4 in Figure 7). After deleting a few Czech surnames (mostly connected by a single link to one of the foreign names shown) the figure almost exclusively contains typical members of five groups of names that are exemplars of Vietnamese, Ukrainian, Chinese (Chen, Lin, Li, Xu, Zhou), Roma, and some German origin surnames. While the frequent spatial co-occurrence of the last two groups was already outlined above, the finding of proximity between other groups is both new and interesting. The fact that these ethnically specific groups (or their exemplar surnames) occupy a similar position in the Czech surname space (and cannot be found elsewhere in the network) demonstrates that they differ from the Czech majority population and reveal similarity in their spatial behaviour. At the same time, however, members of these groups still keep a considerable degree of specificity as suggested by the existence of more or less recognizable clusters of these communities.

## Conclusions and Possible Applications

This paper is premised on the observation that the majority of Czech surnames demonstrate unique geographic distributions that combine to create regionally distinct surname compositions. This was extended to suggest that surnames with similar geographic patterns are more likely to be related in some way (as a cultural attribute) than those with very different distributions. Through the application of suitable measures of spatial co-occurrence, the extent of revealed relatedness between individual pairs of surnames was quantified. The focus here was not an intensive examination of the proximities between particular surnames; instead, the ultimate goal was to understand the aggregate pattern of the Czech surname space, anticipating that some innovative insights about the Czech population structure can be gathered in this way too.

We conceptualized and represented the Czech surname space as an undirected network of surnames linked by their pairwise revealed relatedness. This approach demonstrated the utility of network representations and techniques in the context of surname data that appears to share several properties often attributed to other complex networks. These include a relatively inhomogeneous structure, considerably skewed degree distribution, and multi-layered composition determined by a highly right skewed frequency distribution of surnames. This falls hand in hand with a pronounced hierarchy regarding spatial scales on which the concentrations of these surnames occur.

Indeed, the results confirmed a great deal of correspondence between the macro-structure of the Czech surname space and the main cultural and historical macro-divisions of the Czech population. The more detailed analysis has proved useful in offering numerous more nuanced insights about Czech population structure such as the identification of less known secondary divisions or specific clusters of surnames. It has also been shown that the inspection of network parameters such as density or the mean degree between particular parts of the surname space can be used for comparing the extent of homogeneity and stability between different populations or their parts.

This work represents an initial foray with a wide range of further applications. Importantly, most of the methods presented here are scalable so that they can be analogously used for analyzing different spatial systems or different parts or regions within one spatial system.

Another possible application is related to the identification of the clusters of high degree surnames found in the cores of the Czech surname network. These “hub” surnames can be regarded as the most typical and stable exemplars of their respective parts of the surname space, and together, can be considered a backbone of the Czech surname space. The identification of these most typical and stable surnames (and mapping of the main areas of their concentrations) offers a valuable tool for population geneticists, who for example are seeking to optimise their sampling design. Such names can indicate aspects of population structure, such as rates of population turnover, that may be more or less conducive to genetic sampling. For example, it would be ineffective to target a population group comprising large numbers of migrants if trying to characterise the genetic attributes of the historic population of the specific area in which the migrants reside. In this sense, our study provides another example of promising potential for integration of geography and genetics [24].

Although our analysis utilized current cross-sectional data, there exists a potential for insights into long-term population processes. This is most evident in relation to the enduring spatial stability of a majority of Czech surnames in spite of a long history of population movements. Such movements, therefore, appear to have only marginal impacts on regional surname structure. The exceptions are rare but notable as they point to the radical population changes associated with the expulsion of Germans from the post-war Czechoslovakia and subsequent resettlement of the formerly largely German speaking areas. This presents further avenues for research that could, for example, focus on the separate surname network for the former German areas and compare its parameters with the rest of the country. If an appropriate theoretical framework is applied, this one-time population shock can be considered as a kind of “natural experiment” and the persistence and resilience of the affected surname system may be examined using the methodology described above.

Another notable feature perturbing the stability of the Czech surname space is the specific spatial behaviour of various minority population groups including international migrants. Although, in quantitative terms, these groups still represent a minor part of the Czech population, this study has shown that they are a well-delineated segment. On this basis, our analysis may be considered a tool for the classification of surnames into ethnic groups based solely on their spatial characteristics. It can be thus considered as an alternative to existing approaches to name-based ethnicity classifications that harness pre-existing ethnic categories of surnames [25]. The combination of these two approaches therefore offers a promising avenue of future research in which a classification is created and validated based on a series of inductive spatial and non-spatial surname metrics.

In summary, this study sought to demonstrate the applicability of a new approach to surname research as a means of revealing the underlying surname structure of a country, in this case the Czech Republic. It is our hope that the perspective and methodology adopted here can serve as a template for similar studies in other countries and facilitate further interdisciplinary research in this area.

## Supporting Information

Figure S1 **Ethno cultural differentiation of Czechia.**
(TIF)

Figure S2 **High resolution version of Czech surname space based on surnames co-occurrence in micro-regions.**
(PDF)

Figure S3 **The Czech surname space based on surnames co-occurrence in micro-regions: node size proportional to the degree of particular surnames.**
(PDF)

Figure S4 **Surname degree versus surname population size.**
(PDF)

Figure S5 **High resolution version of Czech surname space based on surnames co-occurrence in municipalities.**
(PDF)

Text S1 **Tests of behaviour of** ***J_i,j_*** **and** ***D_i,j_*** **with respect to differing population size.**
(PDF)

Text S2 **Ethno-cultural differentiation of Czechia and main migratory trends over the second half of 20^th^ century.**
(PDF)
