# Supplementary figures and images for: The Surname Space of the Czech Republic: Examining Population Structure by Network Analysis of Spatial Co-Occurrence of Surnames
Source: PLoS One. 2012 Oct 31;7(10):e48568. doi: 10.1371/journal.pone.0048568 (PMC3485322; doi:10.1371/journal.pone.0048568)

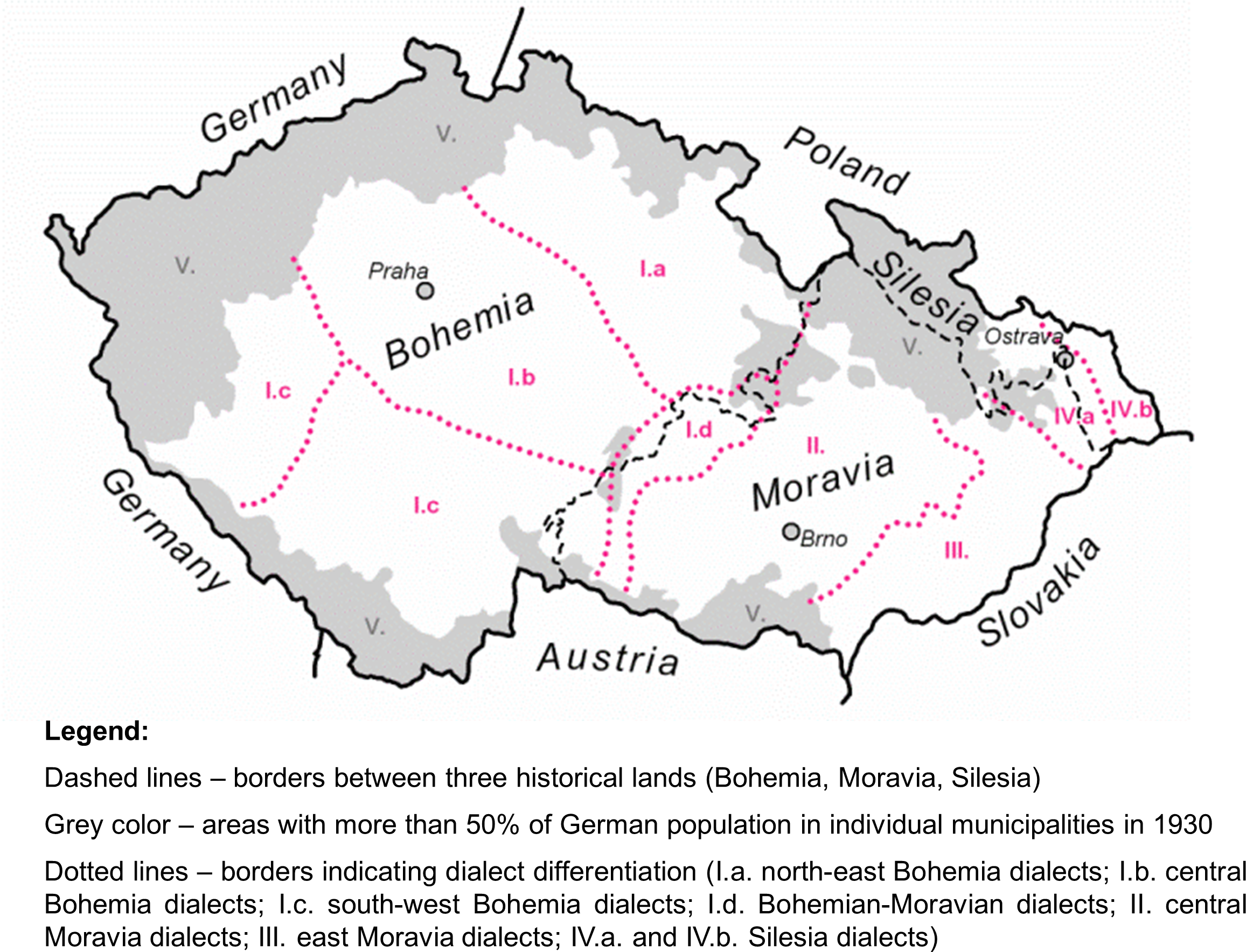

Supplement: Figure S1 — Ethno cultural differentiation of Czechia. (TIF) [file pone.0048568.s001.tif]

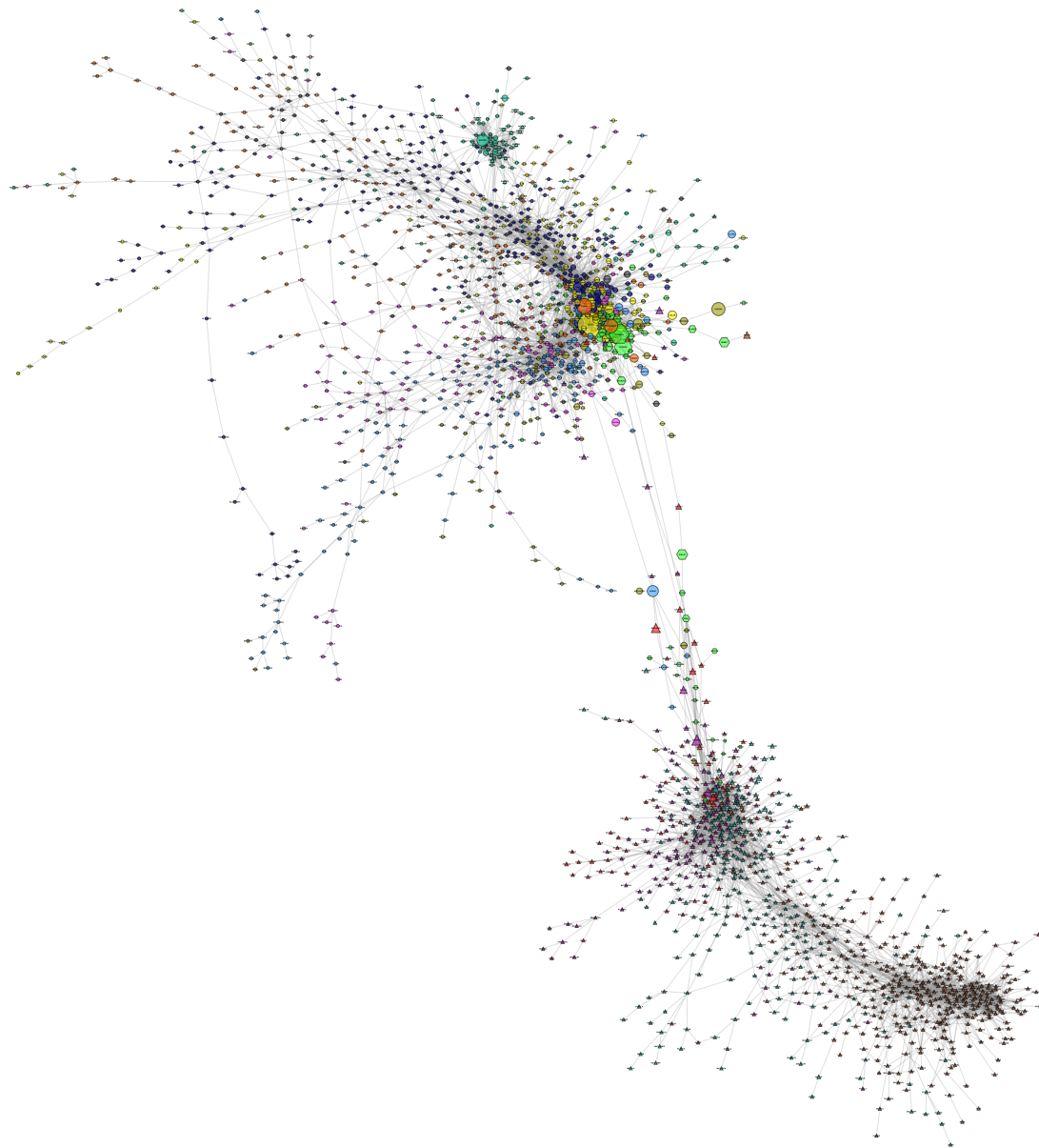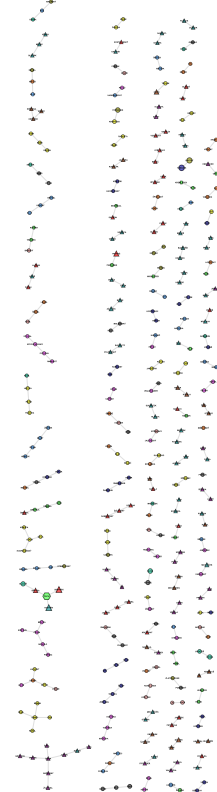

Supplement: Figure S2 — High resolution version of Czech surname space based on surnames co-occurrence in micro-regions. (PDF) [file pone.0048568.s002.pdf]

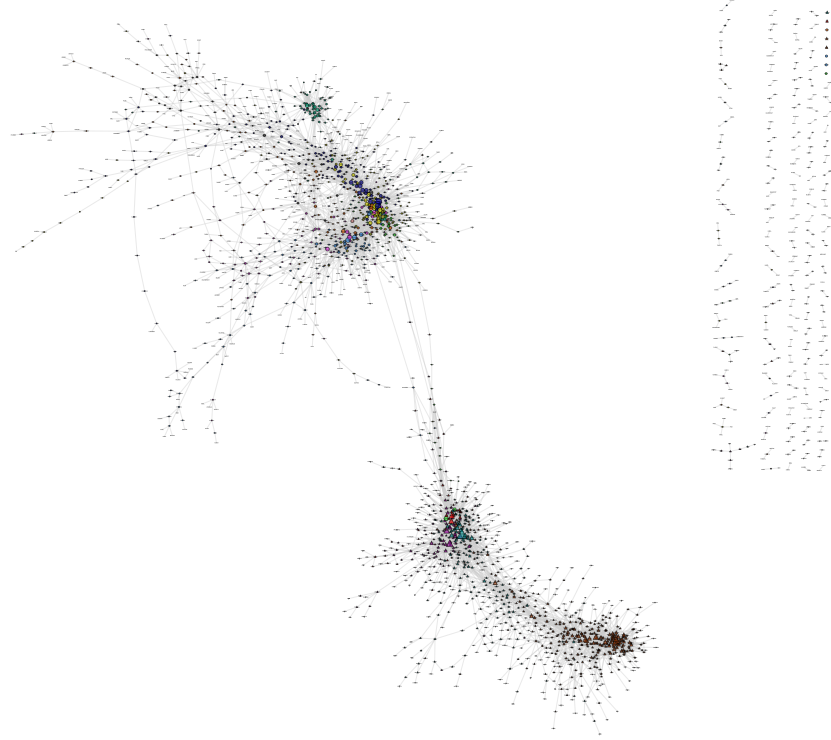

Supplement: Figure S3 — The Czech surname space based on surnames co-occurrence in micro-regions: node size proportional to the degree of particular surnames. (PDF) [file pone.0048568.s003.pdf]

**Figure S4**

*Surname degree versus surname population size (based on surname network in Figure 2)*

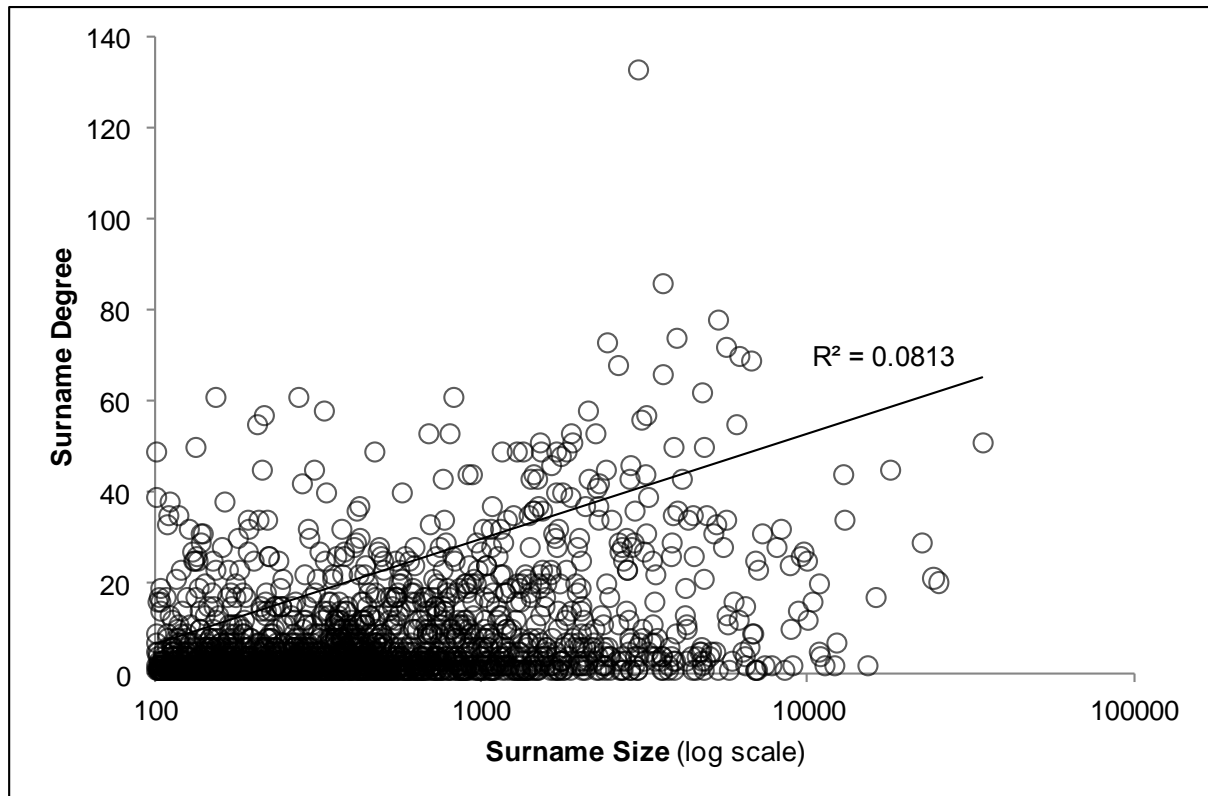

Supplement: Figure S4 — Surname degree versus surname population size. (PDF) [file pone.0048568.s004.pdf]

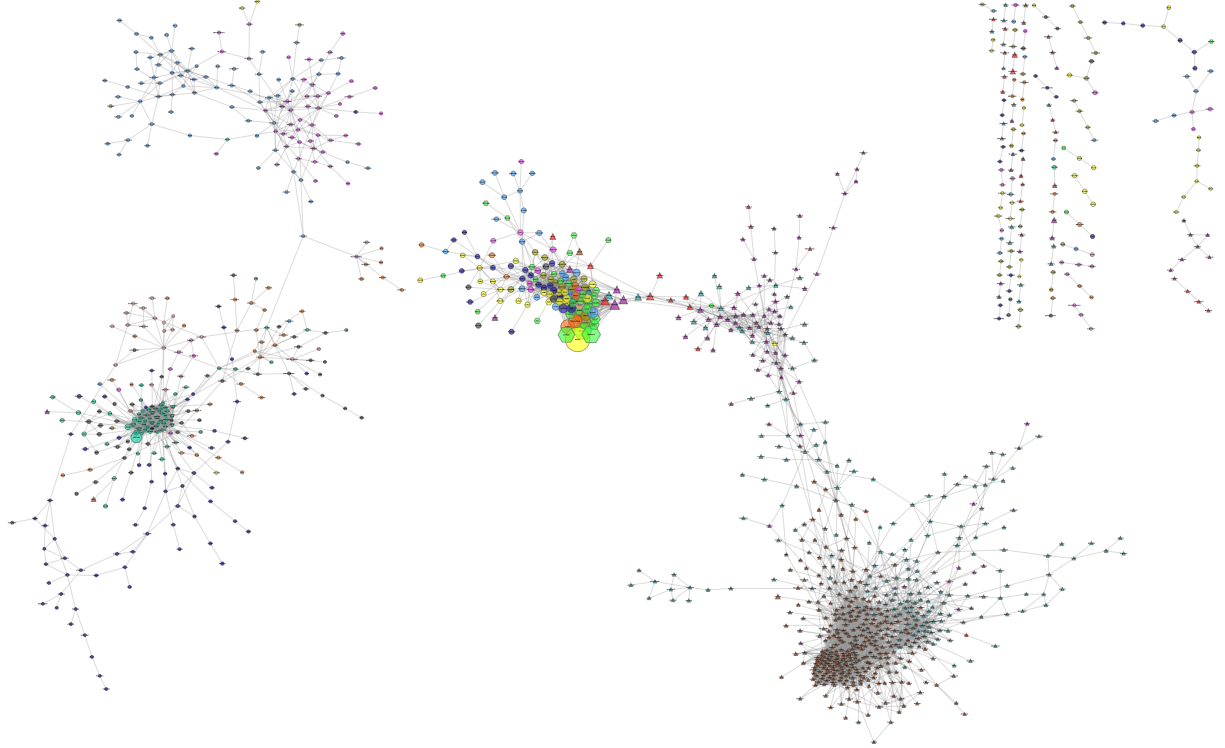

Supplement: Figure S5 — High resolution version of Czech surname space based on surnames co-occurrence in municipalities. (PDF) [file pone.0048568.s005.pdf]
