# Supplementary material for: The Surname Space of the Czech Republic: Examining Population Structure by Network Analysis of Spatial Co-Occurrence of Surnames
Source: PLoS One. 2012 Oct 31;7(10):e48568. doi: 10.1371/journal.pone.0048568 (PMC3485322; doi:10.1371/journal.pone.0048568)

Text S1

Tests of behaviour of $J_{i,j}$ and $D_{i,j}$ with respect to differing population size

The appropriateness of the Jaccard ($J_{i,j}$) and Dice ($D_{i,j}$) measures has not been tested with surname data. We therefore sought to establish the possible impacts of differing population sizes of individual surnames. We generated pseudorandom dataset consisting of 13 subpopulations (people with 13 different surnames) with the size ranging from 50 to 35,000 individuals, which roughly corresponds to the actual range of surname sizes in our dataset on Czech surnames. Each of these sub-populations was randomly allocated into 206 groups (regions) when the probability that an individual would fall into a group was fixed according to the actual shares of 206 Czech regions in the total population. We then calculated the Jaccard and Dice pairwise measures between the subpopulation with the size of 500 and each of the 12 remaining groups. This procedure was then repeated 1,000 times with repeatedly generated data and the average results were calculated.

Mean values based on 1,000 repetitions

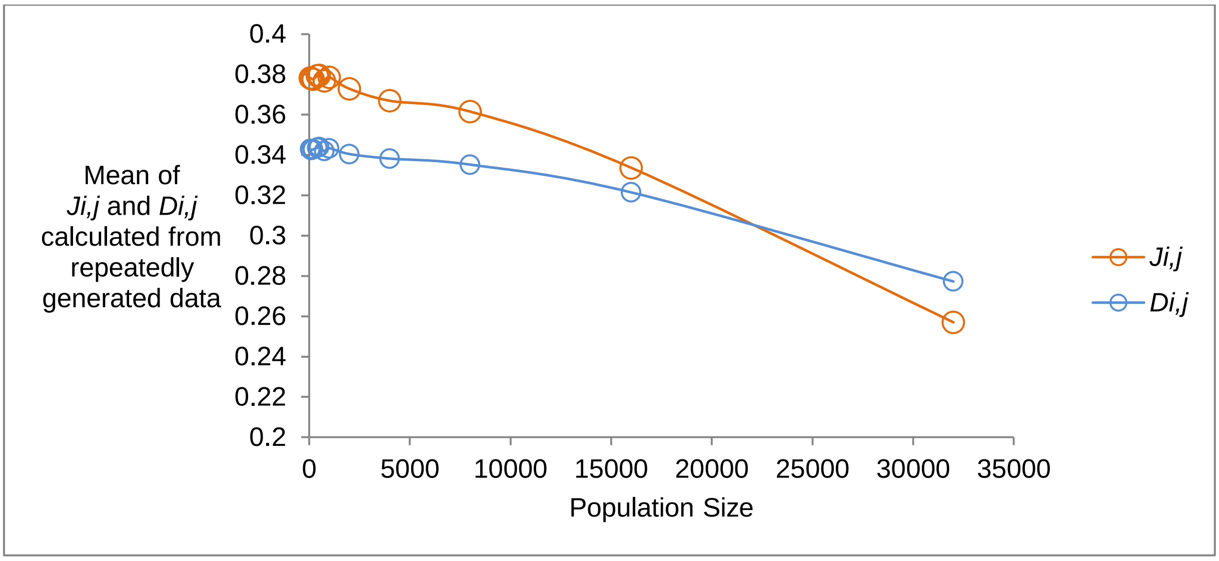

Coefficient of variation of results based on 1,000 repetitions

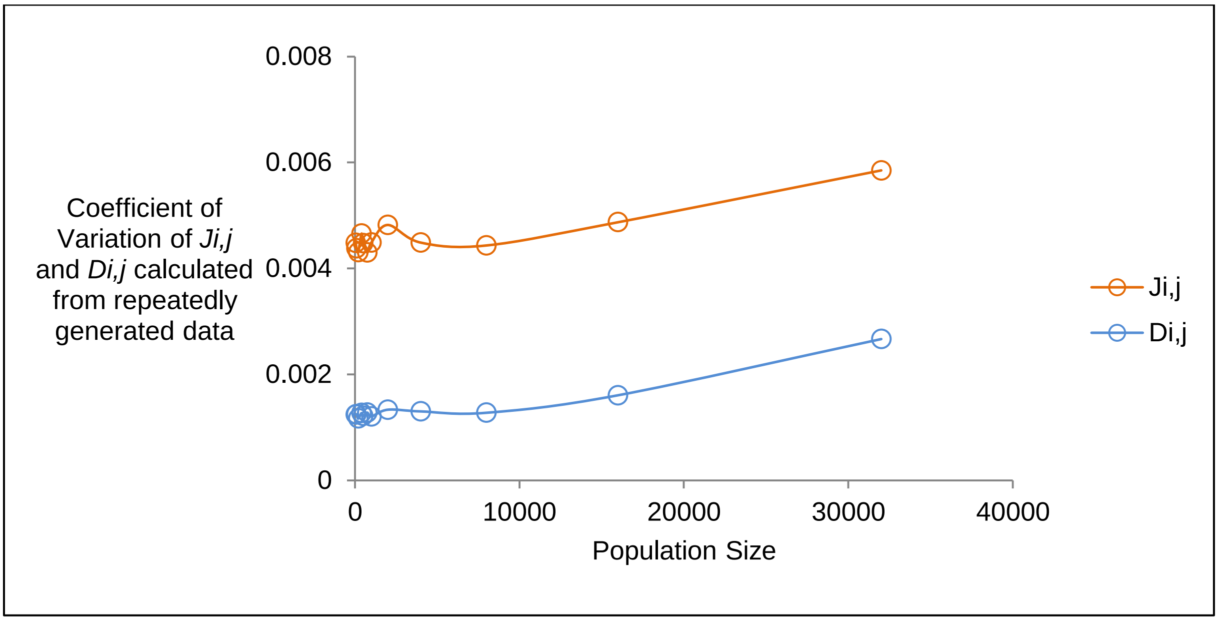

Supplement: Text S1 — Tests of behaviour of Ji,j and Di,j with respect to differing population size. (PDF) [file pone.0048568.s006.pdf]
