# Supplementary material for: The Surname Space of the Czech Republic: Examining Population Structure by Network Analysis of Spatial Co-Occurrence of Surnames
Source: PLoS One. 2012 Oct 31;7(10):e48568. doi: 10.1371/journal.pone.0048568 (PMC3485322; doi:10.1371/journal.pone.0048568)
Supplement: Text S2 — Ethno-cultural differentiation of Czechia and main migratory trends over the second half of 20th century. (PDF) [file pone.0048568.s007.pdf]

<u>Supplementary information – Text S2</u>

Ethno-cultural differentiation of Czechia and main migratory trends over the second half of 20th century

Despite a relatively small area, Czechia is a diverse country partitionable into partially overlapping regions distinguishable on several geographical scales (eg Hampl et al. 1987; Siwek and Bogdová 2007). At the highest spatial level, boundaries between three historical lands reflect a major cultural division of the country (see Figure S1). The three lands include Bohemia (to the west; approximately 2/3 of both total population and area), Moravia (to the east; around 1/3 of total population), and Silesia (the north-east; approximately 1/10 of total population). From the three lands, the smallest Silesia, and particularly its very south-eastern part, is especially unique both regarding political-historical and ethno-cultural characteristics including many specific variants of Czech and Polish surnames (Siwek 2006, 2008). The main division into the three historical lands is also connected to linguistic differentiation of the country with three main dialectical groups in terms of Czech, Moravian, and Silesian group separated by fuzzy borders and transient areas. Several more specific dialectical areas are also distinguishable at more detailed spatial levels (Balhar, Jančák et al. 1992-2005; Figure S1).

Being in the middle of continental Europe, the Czech lands have never been isolated from external cultural influences. After the collapse of Austrian-Hungarian Empire, newly born Czechoslovakia was established in 1918 as a multi-ethnic state with Czech part comprising approximately 2/3 of Czechs, almost 1/3 of Germans (more than 3 million before the WW2) and smaller but not insignificant shares of Polish, Jewish and Roma population. In spatial terms, Germans represented an overwhelming majority of inhabitants of Sudetenland, a belt along the

contemporary northern, western, and southern border of the country (see Figure S1). However, the genocide of Jews and Roma population during the Second World War and especially the post-war expulsion of Germans (2.8 millions of people, mostly during 1945-1946) dramatically homogenized Czech population structure. Together with following resettlement of formerly German areas, this disruptive event brought almost complete replacement of surnames in the former German population border areas (see e.g. Kučera 2007; Chromý et al. 2009)

Another major migratory process that shaped population structure and surname distribution after the WW2 were massive inflows of new work force into coal and heavy industry zones located mainly in north Moravia (Ostrava region) and north and west Bohemia that were taking place approximately until the mid-1960s (Čermák 1999). As a part of both the resettlement of former German areas and industrialization-led population movements, many immigrants from Slovakia and other countries came including also specific groups of Roma population (Davidová 1995).

In addition, from the above mentioned population movements, there has also been a long-term process of urbanization including a natural tendency of population concentration over the most of the 20th century (Hampl et al. 1987). However, this general trend had only gradual effects on surname structure. Moreover, permanent internal migration has been generally low in more recent decades and predominantly on short distances within the same districts (Čermák 1999). According to the official records, only 2.5% (254,147 people) of the Czech population in 1992 left the place of their permanent residence over the past decade of 1992-2011. As such, with the notable exceptions of the above mentioned radical changes that took place in the border areas, the spatial distribution of traditional surnames in Czechia can be considered as being spatially stable.

Given growing intensity of immigration from abroad (e.g. Drbohlav 2011), traditional surnames have been increasingly supplemented by foreign ones. Although the share of foreigners staying permanently in Czechia on a legal basis is still relatively low (around 0.5% in 2011), these groups are interesting for the present study because they often tend to be spatially concentrated. In Czechia this is the case for Vietnamese (55,585 of officially registered people in 2011) with concentration in western and north-western border areas of the country, for Russian immigrants concentrated in a few enclaves such as Karlovy Vary, and to some extent also for the largest immigrant group from Ukraine (106,040 of legally staying immigrants in 2011) who tend to be more prevalent in areas with higher demands for labour (Novotný et al. 2007; Čermák and Janská 2011).

References:

Balhar J. - Jančák, P. et al. (1992-2005): Český jazykový atlas (The Atlas of Czech Language). 5 Vol, Praha, Academia.

Čermák, Z. (1999): Distinctive features of migration in the Czech Republic as part of the transformation of Central European countries. In: M. Hampl (ed.). Geography of Societal Transformation in the Czech Republic. Praha, DemoArt, pp. 209–222.

Čermák, Z., Janská, E. (2011): Rozmístění a migrace cizinců jako součást sociálnĕgeografické diferenciace Česka. Geografie, 116, 4, 422–439.

Chromý, P., Kučrová, S., Kučera, Z. (2009): Regional identity, contemporary and historical regions and the issue of relict borders – the case of Czechia. In: Heffner, K. (ed.): Historical Regions Divided By the Borders: Cultural Heritage and Multicultural Cities. Regions and Regionalism, No. 9, vol. 2. Państwowy Instytut Naukowy – Instytut Śląski w Opolu, Opole, s. 9–19.

Davidová, E. (1995): Romano drom – Cesty Romů. Olomouc, Vydavatelství Univerzity Palackého.

Drbohlav, D. (2011): Imigrace a integrace cizinců v Česku: několik zastavení v její migrační proměně z Davida na téměř Goliáše. Geografie, 116, 4, 401–421.

Hampl, M., Gardavský, V., Kühnl, K. (1987): Regionální struktura a vývoj systému osídlení ČSR. Praha, Univerzita Karlova

Kučera, Z. (2007): Zanikání sídel v pohraničí Čech po roce 1945 – základní analýza. Historická geografie, 34, 317–334.

Novotný, J., Čermáková, D., Janská, E. (2007): Rozmístění cizinců a jeho podmiňující factory: pokus o kvantitativní analýzu. Geografie, 112, 2, 204-220.

Siwek, T. (2006): Polacy, Ślązacy, Zaolziacy i Czesi polskiego pochodzenia. In: Leoński, J. – Wątróbski, L. (eds.): Diaspora I. Socjologiczne, kulturowe, językowe i historyczne szkice o polskiej diasporze. Rozprawy i studia 606, Uniwersytet Szczeciński, Szczecin, 143-150.

Siwek T. (2008) Nazwiska jako indykator pochodzenia ludności miasta Český Těšín. In: D. Świątek, M. Bednarek, P. Siłka (Ed.) Współczesne problemy badawcze geografii polskiej – geografia człowieka. Dokumentacja Geograficzna IGiPZ PAN, 36, 196-201.

Siwek, T., Bogdová, K. (2007): České kulturně historické regiony ve vědomí svých obyvatel. Sociologický časopis, 43, 4, 1039-1053.
